# Physiologic and anesthetic predictors of false-positive motor-evoked potential alerts during pediatric spine surgery

**DOI:** 10.1007/s43390-026-01401-2

**Published:** 2026-05-15

**Authors:** Alex La Poche, Austin Montgomery, John Sestito, James Hutchinson, Thomas Romanelli, Andrew Franklin, Jeffrey Martus, Gregory Mencio, Craig Louer

**Affiliations:** 1 Department of Orthopedic Surgery, Vanderbilt University Medical Center, Nashville, TN USA; 2 Department of Neurodiagnostics, Vanderbilt University Medical Center, Nashville, TN USA; 3 Department of Anesthesiology, Vanderbilt University Medical Center, Nashville, TN USA; 4 Department of Orthopedic Surgery, SUNY Downstate College of Medicine, Brooklyn, NY USA

**Keywords:** Intraoperative neuromonitoring, Motor evoked potentials, False positive alerts, Pediatric spinal deformity surgery, Bispectral index (BIS), Total intravenous anesthesia (TIVA)

## Abstract

**Purpose:**

False-positive transcranial motor-evoked potential (tcMEP) alerts during pediatric spinal deformity surgery can delay or alter surgical management. This cohort study evaluated anesthetic and physiologic predictors of false-positive alerts under standardized total intravenous anesthesia (TIVA).

**Methods:**

All pediatric spine deformity surgeries with intraoperative tcMEP alerts (June–November 2024) at a single tertiary center were identified from a quality registry. Alerts were classified as false positive (FP), true positive (TP), or true negative (TN) by intraoperative assessment and postoperative outcomes. Standardized total intravenous anesthesia with bispectral index (BIS) and electroencephalography (EEG) monitoring was used. Physiologic and anesthetic variables were retrospectively extracted and compared using appropriate parametric and nonparametric tests (*p* < 0.05).

**Results:**

Of 99 patients during the study period, 15 experienced tcMEP alerts, yielding 631 timepoints: 80 FP, 13 TP, and 538 TN. BIS values were significantly lower during FP alerts (28.8 ± 14.5) than TP (53.6 ± 9.9) or TN (40.1 ± 12.9) (*p* < 0.001). EEG suppression occurred in 36.4% of FP timepoints but in 0% of TP and 0.9% of TN events (*p* < 0.001). Receiver operating characteristic (ROC) analysis showed that BIS had outstanding discrimination between false-positive and true-positive alerts (AUC 0.924, 95% CI: 0.823–1.00).

**Conclusion:**

Periods of BIS suppression, in the setting of stable hemodynamics and anesthetic delivery, strongly correlate with false-positive tcMEP alerts. Incorporating BIS into intraoperative decision-making may help distinguish false positives from true neurologic injury, reducing unnecessary interruptions in pediatric spinal deformity surgery.

## Introduction

Intraoperative neuromonitoring (IONM) has become a critical tool in pediatric spine surgery, with transcranial motor-evoked potentials (tcMEPs) playing a central role in the early detection and prevention of neurologic injury. TcMEPs provide real-time assessment of motor pathway integrity during high-risk maneuvers such as deformity correction, osteotomies, or screw placement [1]. Signal degradation temporally associated with a surgical insult may indicate evolving spinal cord injury, allowing for timely intervention and neurological preservation [2].

Despite its clinical value, IONM is susceptible to false-positive (FP) alerts, defined as tcMEP decreases that are not related to spinal cord compromise. These FP alerts can lead to unnecessary operative delays, redundant imaging, altered surgical strategy, and heightened anxiety for the surgical team and family [3, 4]. Recent work suggests that factors such as anesthetic depth, cerebral perfusion, and systemic physiology may modulate tcMEP responsiveness, complicating the interpretation of alerts in the absence of a clear surgical cause [5].

Anesthetic management is a key determinant of tcMEP reliability. Total intravenous anesthesia (TIVA), typically employing propofol and short-acting opioids, is widely recommended to reduce interference from inhalational agents [6]. However, even within TIVA protocols, variability in depth of anesthesia, reflected by bispectral index (BIS) and EEG suppression patterns, may contribute to neurophysiologic changes that mimic true alerts [7, 8].

The incidence and impact of FP tcMEP events are underreported in the pediatric spine deformity literature. To improve understanding and awareness of these alerts, we performed a timepoint-level analysis of intraoperative physiologic and anesthetic variables surrounding tcMEP alerts during pediatric posterior spinal fusion. We hypothesized that FP alerts would be associated with deeper anesthesia, as evidenced by decreased BIS, increased EEG suppression (interburst interval (IBI) > 0), and longer IBI duration, compared to both TP alerts and non-alerting tcMEP events (true negatives). Our goal was to quantify these associations, characterize the operative delays and workflow disruptions associated with FP alerts, and identify markers that could aid intraoperative differentiation between true and false neuromonitoring alerts.

## Methods

**Fig. 1:**
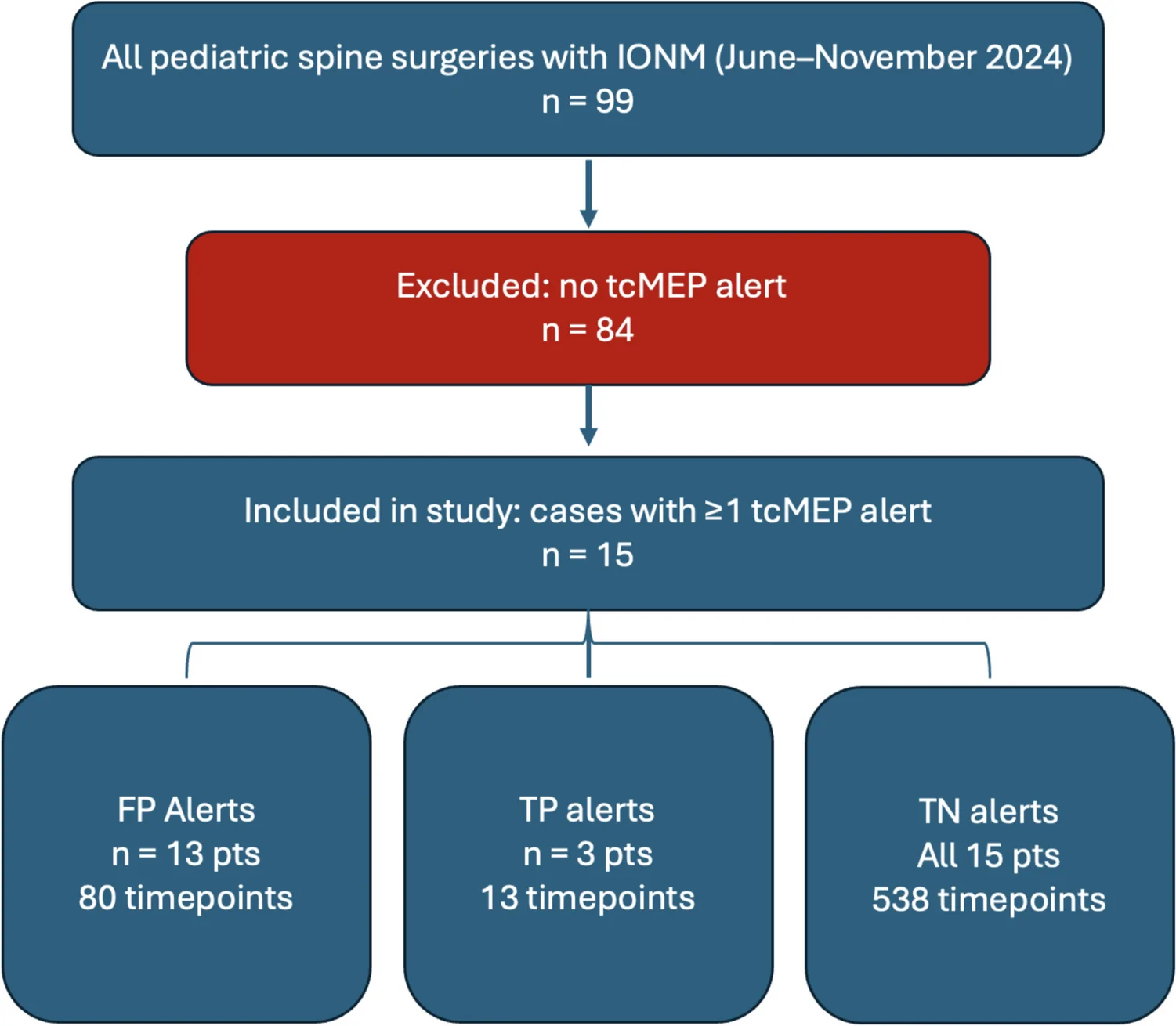
Study cohort selection and timepoint classification. Flow diagram illustrating patient selection and tcMEP timepoint classification. Of 99 pediatric spine surgeries performed during the study period, 15 cases with ≥ 1 tcMEP alert were included. These yielded 631 intraoperative timepoints, categorized as false positive (FP), true positive (TP), or true negative (TN) based on intraoperative assessment

This is a prospective, registry-based study conducted at a single tertiary center, with retrospective analysis of intraoperative neuromonitoring alerts in a cohort undergoing posterior spinal fusion between June and November 2024. Only cases with transcranial motor-evoked potential (tcMEP) alerts were included (Fig. 1). TcMEPs were obtained at key surgical stages (exposure, osteotomies, screw insertion, correction), with timing tailored to the case by each surgeon. During this period, alerts were reviewed in real time by the surgical and neuromonitoring teams, and the presence or absence of a surgical cause was documented. Intraoperative data were subsequently extracted and analyzed. All cases were monitored using a multimodal IONM protocol including tcMEPs, SSEPs, free-run EMG, and triggered EMG; tcMEPs were not monitored in isolation.

TcMEP timepoints were classified as FP, TP, or TN in real time during surgery by the operating surgeon and neuromonitoring team based on intraoperative context, specifically whether a surgical maneuver was performed that could explain IONM changes. These determinations were documented contemporaneously in neuromonitoring logs and operative records. Anesthetic depth variables, including BIS values and EEG suppression metrics, were not used to determine alert classification. Postoperative neurologic examinations corroborated intraoperative alert classification. When tcMEP changes were temporally associated with an at-risk surgical maneuver, alerts were treated as true positives and prompted immediate corrective action with use of our institutional IONM checklist. Without a plausible surgical cause, alerts were interpreted as false positives and managed per the treating surgeon’s determination, at times bypassing aspects of the IONM rescue checklist. Alerts involving tcMEP decrease or loss without a clear surgical cause or deficit were considered FP timepoints. Signal loss linked to a plausible insult or neurologic change was considered a TP event. Stable tcMEPs were considered TN timepoints.

**Fig. 2:**
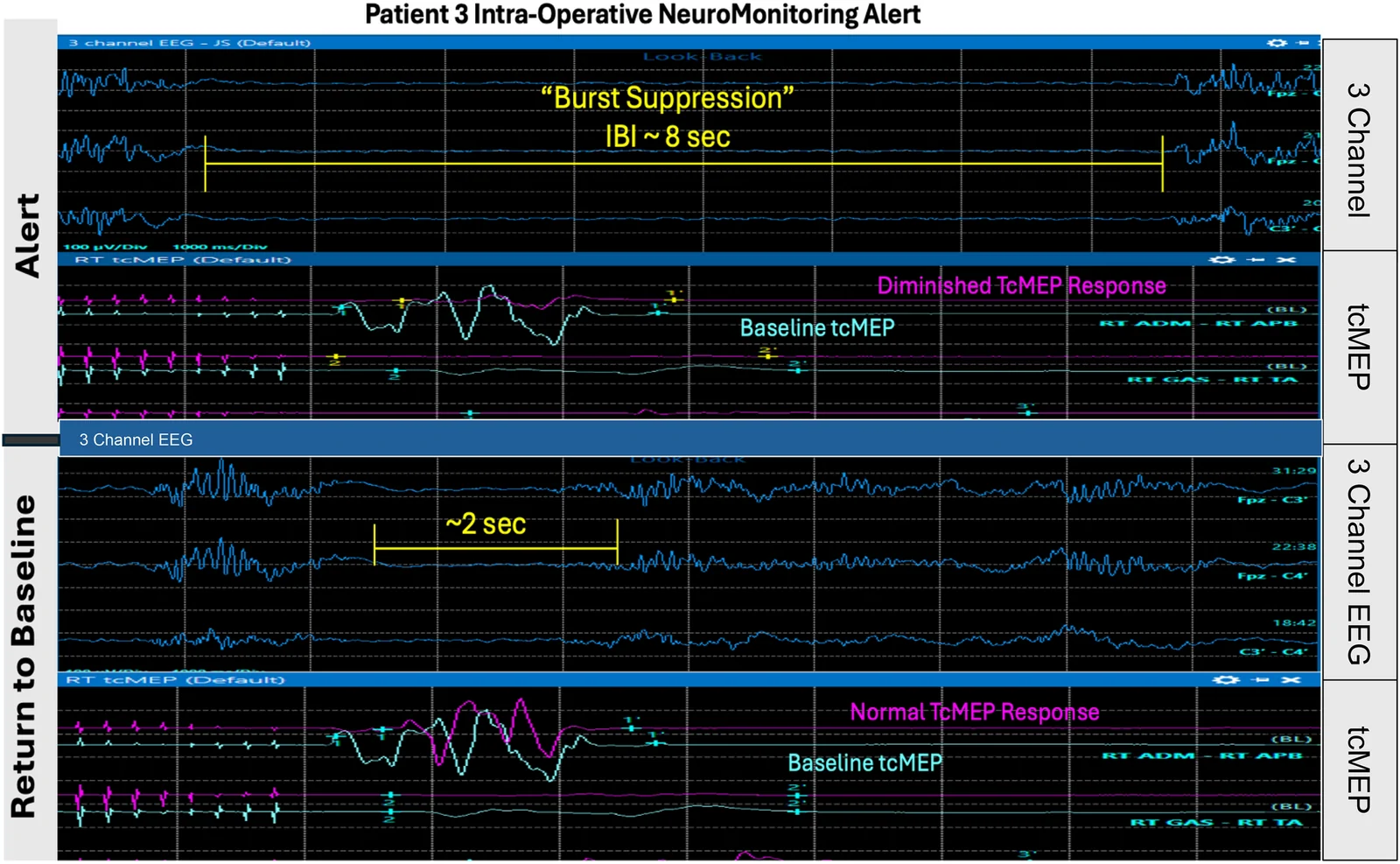
EEG interburst interval (IBI) Tracing Example. Three-lead EEG and corresponding tcMEP recordings from a representative case. The top panel (“Alert”) shows burst suppression, with critical suppression between bursts representing an interburst interval (IBI) of ~ 8 s, accompanied by diminished tcMEP responses. The lower panel (“Return to baseline”) demonstrates recovery of cortical activity with shorter IBIs (~ 2 s) and restoration of normal tcMEP amplitude. This example illustrates that prolonged EEG suppression (long IBI) correlates with decreased motor responsiveness, consistent with anesthetic-related false positives

All cases used a standardized total intravenous anesthesia (TIVA) protocol with continuous propofol and opioid infusion. Inhalational agents were avoided post-induction. Neuromuscular blockade was reversed before tcMEP acquisition. BIS and processed EEG monitoring were recorded throughout. BIS was used as a processed EEG-based indicator of anesthetic depth, consistent with established neurosurgical and anesthetic monitoring literature [9, 10]. EEG suppression was defined by the presence of interburst intervals (IBI > 0), reflecting periods of cortical inactivity between bursts, in accordance with established burst suppression physiology and standardized clinical EEG terminology (Fig. 2) [11, 12].

At each tcMEP timepoint, synchronized anesthetic and physiologic data were extracted, including BIS, EEG suppression (interburst interval > 0), mean IBI duration, mean arterial pressure (MAP), temperature, and infusion rates. Data were compared across groups using appropriate parametric and nonparametric tests (*p* < 0.05). Analyses were performed using Excel, RStudio and GraphPad Prism.

## Results

Of 99 patients undergoing spine surgery with neuromonitoring during the 6-month study period, 15 (10 idiopathic, 2 syndromic, 2 neuromuscular, and 1 congenital) experienced at least one tcMEP alert, yielding 631 intraoperative timepoints; each case also contributed TN timepoints for comparison. Of the 631 events, 80 were classified as FP, 13 as TP, and 538 as TNs. The median number of timepoints per patient was 37 (range 12–76). The highest contributing patient accounted for 12% of total observations and 30% of FP events; FP alerts occurred in 13 of 15 patients. One patient experienced both a TP and an FP alert during the same procedure. Baseline demographic and surgical characteristics did not significantly differ between groups (Table 1).

**Table 1:** Patient demographics and surgical characteristics

	False positive (FP)	True positive (TP)	All patients*	*P* value (TP vs FP)
*Patient-level data*	*n* = *13*	*n* = *3*	*n* = *15*	*1 patient had both TP and FP alerts*
Age	13.5 ± 2.7	13.7 ± 2.5	13.5 ± 2.5	0.995^1^
Height (cm)	153.7 ± 12.0	157.8 ± 10.4	154.5 ± 11.5	0.863^1^
Weight (kg)	46.9 ± 10.3	53.2 ± 7.8	48.2 ± 10.0	0.631^1^
BMI	20.2 ± 5.5	21.3 ± 0.9	20.4 ± 4.9	0.943^1^
Levels fused	12.8 ± 2.9	11.0 ± 5.2	12.4 ± 3.3	0.718^1^
Major Cobb (°)	67 ± 29	105 ± 0.0 *1 case of focal 57 kyphosis not included in analysis of major Cobb angle*	69 ± 32	0.841^2^

^1^*t-*test, ^2^Mann–Whitney test. *All patients had true-negative (TN) timepoints. Values are mean ± SD

**Fig. 3:**
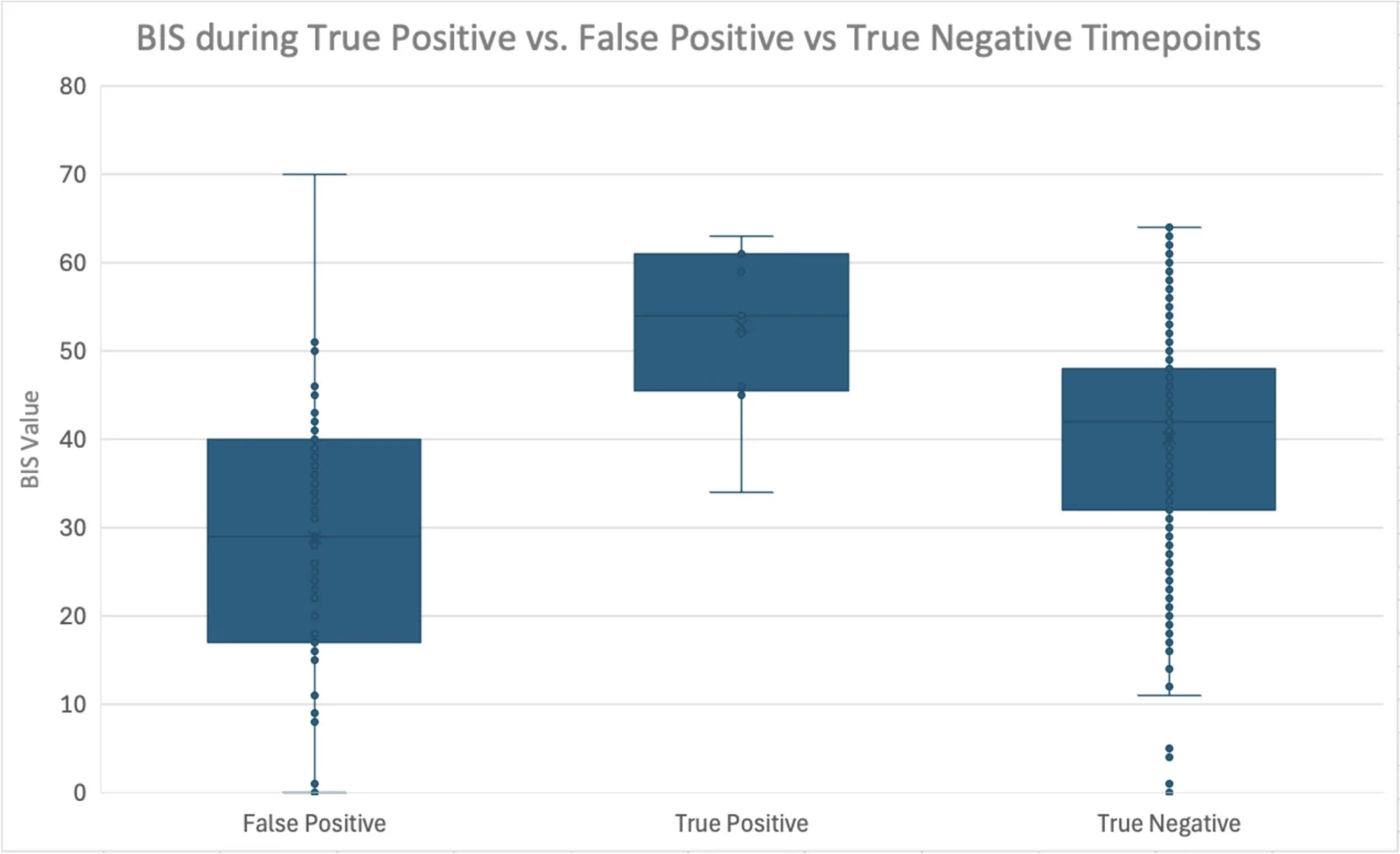
Bispectral Index (BIS) values by alert classification. Box-and-whisker plot comparing BIS across false-positive (FP), true-positive (TP), and true-negative (TN) timepoints. Boxes represent the interquartile range (IQR, 25th–75th percentile), horizontal lines mark the median, and whiskers denote the range within 1.5 × IQR. Individual points represent outliers. BIS values were significantly lower during FP alerts than TP or TN events (*p* < 0.001), indicating deeper anesthesia during false-positive periods

**Fig. 4:**
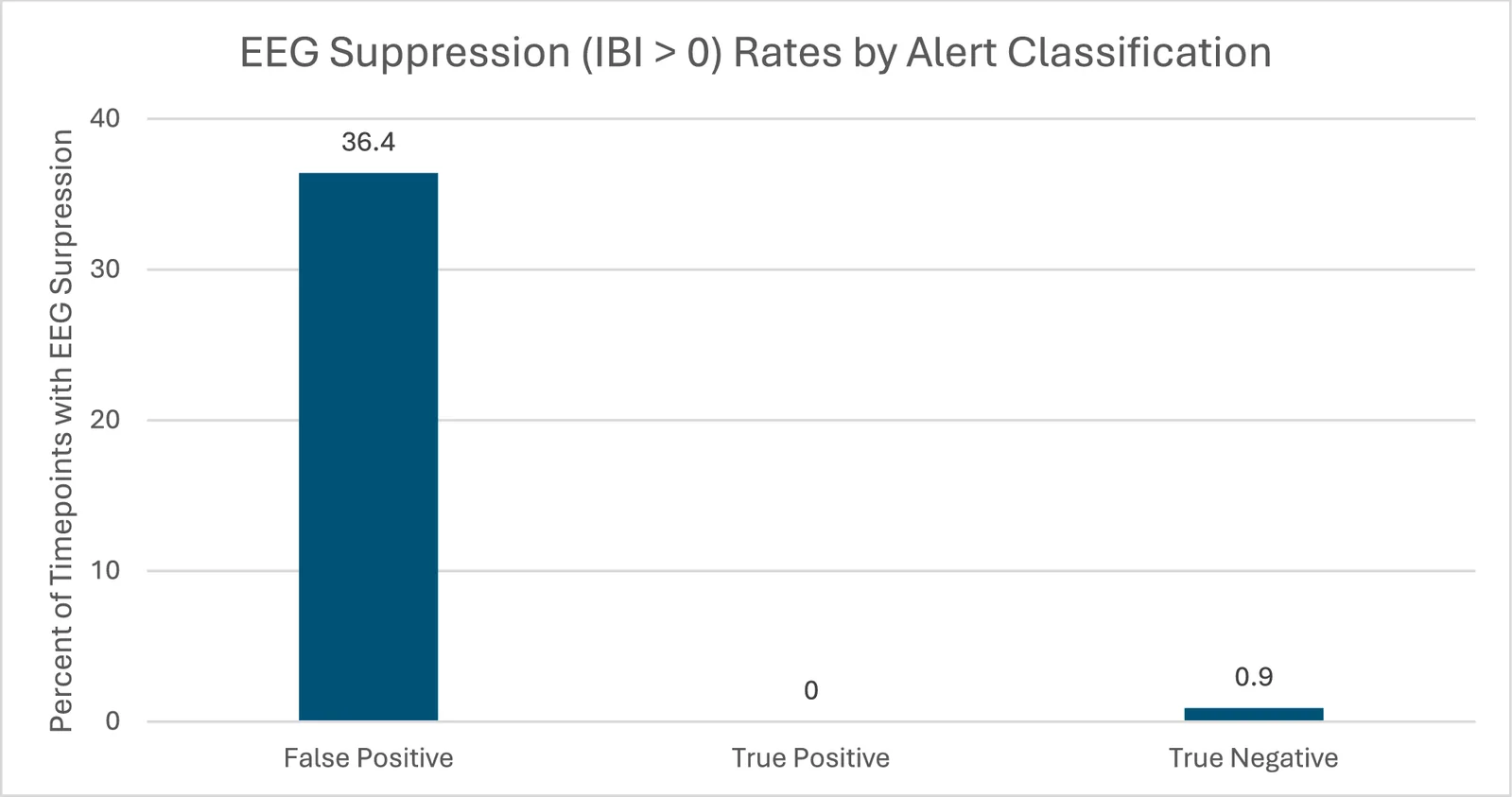
EEG suppression (IBI > 0) rates. Frequency of EEG suppression (IBI > 0) across alert classifications. Bar gaph showing the proportion of timepoints with EEG suppression in FP, TP, and TN groups. Suppression was common during FP alerts but nearly absent during TP and TN timepoints (*p* < 0.001)

At the time of tcMEP alerts, significant differences in neurophysiologic and anesthetic parameters were observed across groups. BIS values were significantly lower during FP alerts (28.8 ± 14.5) than TP (53.6 ± 9.9) and TN (40.1 ± 12.9) timepoints (*p* < 0.001), indicating deeper anesthesia during FP events (Fig. 3). EEG suppression, defined as the presence of interburst intervals (IBI > 0), was present in 36.4% of FP timepoints but was not observed during any TP alerts (0%) and was rare during TN periods (0.9%) (*p* < 0.001) (Fig. 4). No SSEP or EMG alerts occurred during FP events, and bilateral upper extremity tcMEPs were often similarly suppressed, supporting a global anesthetic effect. Similarly, the mean IBI duration was longest during FP alerts (3.3 ± 6.9 s), compared to 0 s in both TP and TN events (*p* < 0.001).

**Fig. 5:**
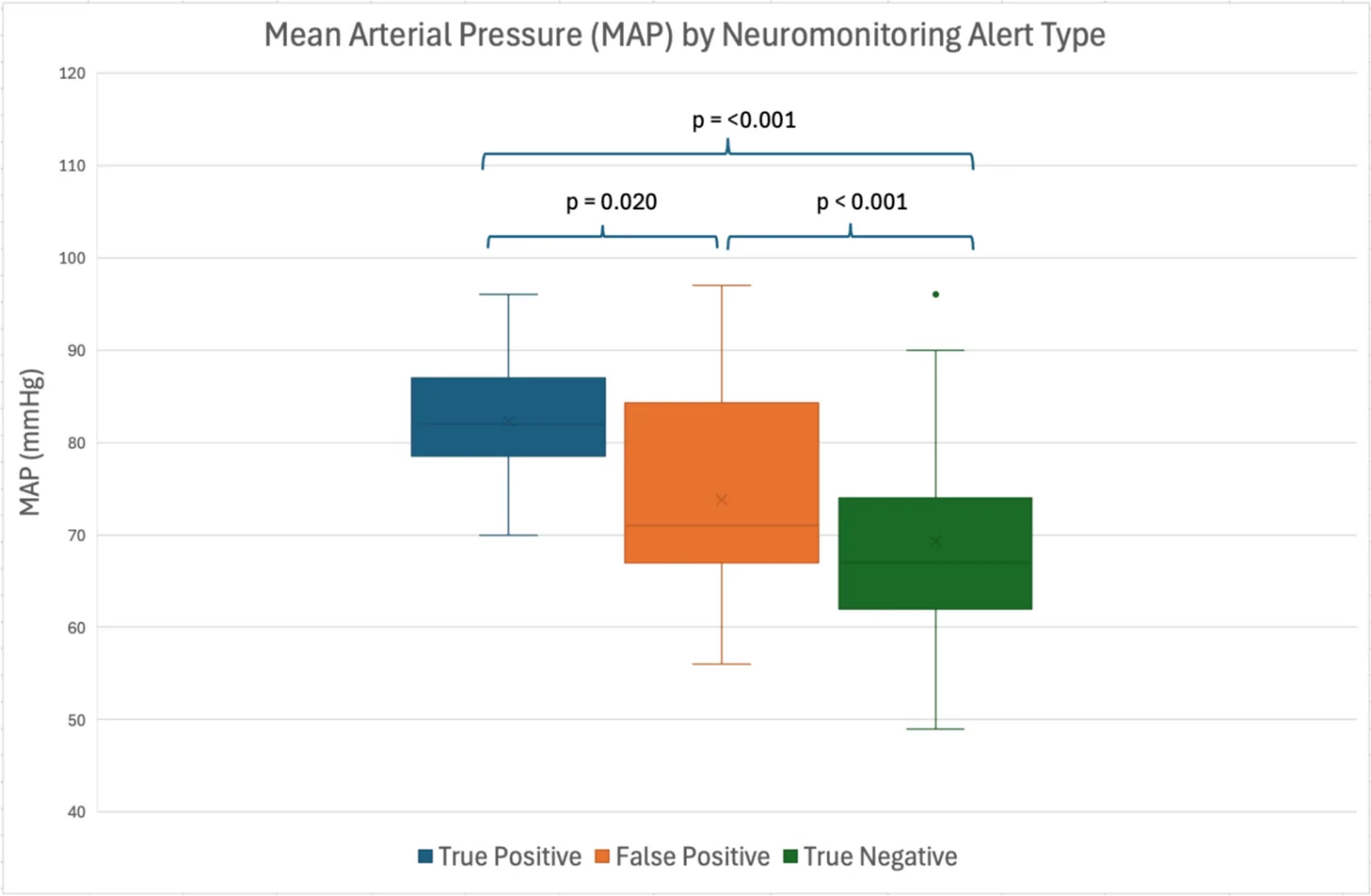
Mean arterial pressure (MAP) by alert classification. Box-and-whisker plot comparing MAP among FP, TP, and TN events. MAP was significantly higher during TP events and lowest during TN periods (*p* < 0.001). Units: mmHg

MAP was paradoxically elevated during FP events (73.8 ± 10.1 mmHg) compared to TN (69.4 ± 17.5 mmHg), though lower than TP events (82.3 ± 7.5 mmHg), with statistically significant differences across groups (*p* < 0.001) (Fig. 5). Body temperature was also significantly lower during FP alerts (35.6 ± 1.4 °C) than during TP (36.9 ± 0.1 °C) and TN (36.4 ± 1.0 °C) timepoints (*p* < 0.001). In contrast, propofol infusion rates did not significantly differ among alert classifications, with average rates of 125.2 ± 43.9 mcg/kg/min during FP, 116.7 ± 28.9 mcg/kg/min during TP, and 137.4 ± 57.8 mcg/kg/min during TN events (*p* = 0.541). Opioid infusion rates, including sufentanil and remifentanil, also showed no significant variation (Table 2).

**Table 2:** Timepoint-level anesthetic and physiologic variables

	False positive (FP)	True positive (TP)	True negative (TN)	All trues (TN + TP)	*P* value (FP vs All Trues)	*P* value (FP vs TP vs TN)
*Timepoints*	*n* = *80*	*n* = *13*	*n* = *538*	*n* = *551*		
Temperature (°C)	35.6 ± 1.4	36.9 ± 0.1	36.4 ± 1.0	36.4 ± 1.0	** < 0.001**^**1**^	** < 0.001**^**2**^
Mean arterial pressure (mmHg)	73.8 ± 10.1	82.3 ± 7.5	69.4 ± 17.5	69.7 ± 19.8	** < 0.001**^**1**^	** < 0.001**^**2**^
Bispectral index (BIS)	28.8 ± 14.5	53.6 ± 9.9	40.1 ± 12.9	38.7 ± 13.7	** < 0.001**^**1**^	** < 0.001**^**2**^
EEG suppression (IBI > 0) [*N* (%)]	8 (36.4%)	0 (0.0%)	1 (0.9%)	1 (0.8%)	** < 0.001**^**3**^	** < 0.001**^**3**^
EEG suppression—IBI (s)	3.3 ± 6.9	0.0 ± 0.0	0.2 ± 1.9	0.2 ± 1.8	** < 0.001**^**1**^	** < 0.001**^**2**^
Propofol infusion rate (mcg/kg/min)	125.2 ± 43.9	116.7 ± 28.9	137.4 ± 57.8	139.8 ± 61.5	0.541^1^	0.736^2^
Sufentanil/remifentanil infusion rate (mcg/kg/min)	0.2 ± 0.1	0.1 ± N/A	0.2 ± 0.1	0.2 ± 0.1	0.438	0.229^2^

^1^Mann–Whitney test, ^2^Kruskal–Wallis test, ^3^Fisher’s exact test. Data are presented as mean ± SD unless otherwise indicated.

Post hoc comparisons with Dunn’s correction confirmed that BIS values were significantly lower in FP alerts compared to TP (*p* < 0.001) and TN (*p* < 0.001) events, with large effect sizes (Cohen’s *d* = –2.00 and –0.83, respectively). EEG suppression also showed statistically significant differences between FP and both TP (*p* = 0.0006) and TN (*p* < 0.001) alerts. These post hoc analyses support the primary hypothesis that neurophysiologic suppression, particularly low BIS values and prolonged IBIs, is a key contributor to FP tcMEP events, independent of surgical manipulation or propofol infusion rate (Table 3).

**Table 3:** Post hoc statistical comparisons—(Dunn’s test with Bonferroni correction)

Variables	FP vs. TP	FP vs. TN	TP vs. TN
Temperature (°C)	**0.0069**	** < 0.001**	0.358
Mean Arterial Pressure (mmHg)	**0.020**	** < 0.001**	** < 0.001**
Bispectral Index (BIS)	** < 0.001**	** < 0.001**	**0.0076**
EEG suppression (IBI > 0) [*N* (%)]	0.481	** < 0.001**	> 0.999
EEG suppression (IBI in seconds)	**0.0006**	** < 0.001**	> 0.999

Adjusted *p* values for pairwise group comparisons following Kruskal–Wallis tests of key physiologic variables

Receiver operating characteristic (ROC) analysis demonstrated excellent discriminatory performance for BIS in distinguishing FP from TP alerts, with an area under the curve (AUC) of 0.924. A BIS threshold of ≤ 44 provided the best accuracy, yielding 87.5% sensitivity and 88.1% specificity. Notably, no true positive alerts occurred with BIS < 34, supporting the use of BIS suppression as a marker of non-pathologic signal loss. In contrast, interburst interval (IBI) showed only modest discrimination (AUC 0.552).

**Fig. 6:**
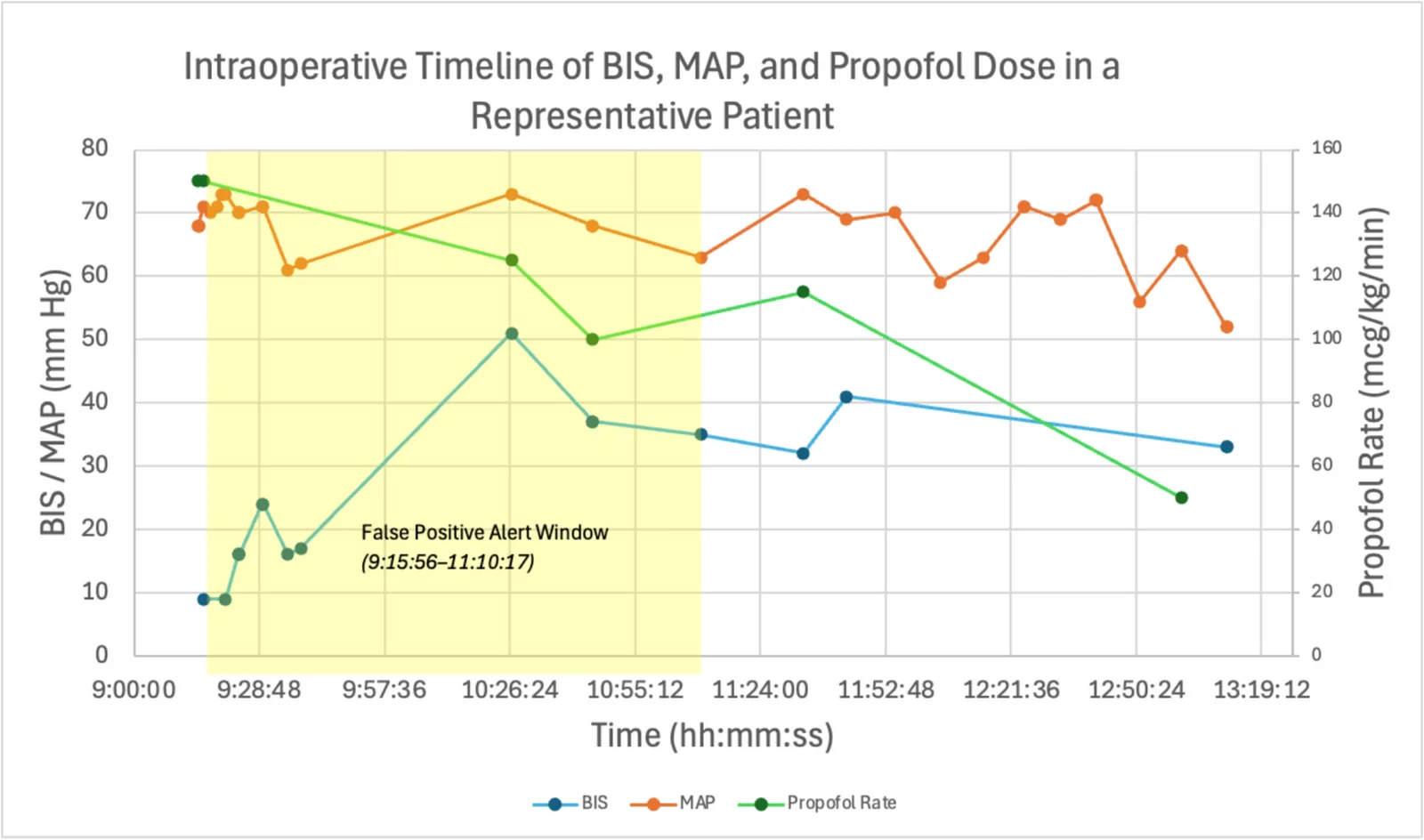
Intraoperative timeline in a representative false-positive (FP) alert case. Intraoperative timeline showing BIS, MAP, and propofol infusion rates over time. A shaded region marks the duration of a false-positive tcMEP alert (~ 9:16–11:10). During this period, BIS remained suppressed (< 50) with stable MAP and consistent propofol infusion (125–150 mcg/kg/min). Baseline MEPs could not initially be obtained, and the case was delayed 25 min before proceeding once the team recognized the anesthetic etiology. Without this awareness, the case likely would have been delayed over two hours or aborted entirely

**Fig. 7:**
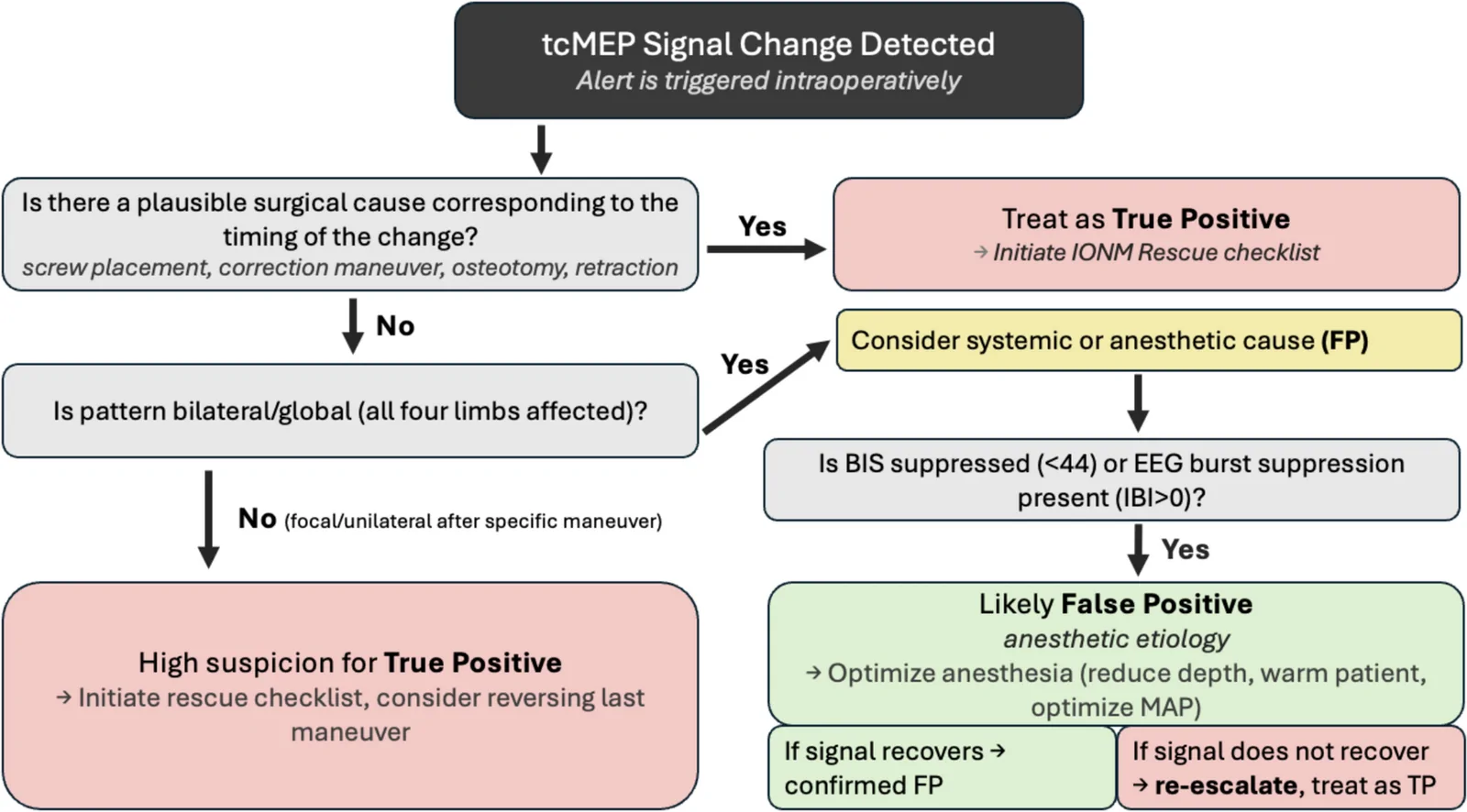
Intraoperative algorithm for interpretation of tcMEP alerts. Decision-making framework for intraoperative interpretation of tcMEP signal changes. Alerts are evaluated based on surgical context, anesthetic depth (BIS and EEG suppression), and signal pattern (global vs focal). The algorithm integrates these factors to guide differentiation between true-positive and false-positive alerts and to inform appropriate intraoperative management

Figure 6 shows a representative FP case. During the alert window, BIS remained suppressed with stable MAP and propofol infusion, supporting an anesthetic rather than surgical cause. Recognition of this pattern limited delay to 25 min; without this awareness, the case may have been significantly prolonged (Fig. 7).

## Discussion

This study examined the etiology of FP tcMEP alerts during pediatric posterior spinal fusion. IONM reduces neurologic risk in spinal deformity surgery, but interpreting tcMEP alerts is challenging, especially without a clear surgical insult. Few studies have examined anesthetic and physiologic contributors at the timepoint level in pediatric cohorts [13, 14]. Our aim was to characterize intraoperative conditions associated with FP alerts and identify patterns supporting real-time differentiation between true and false events.

Our timepoint-level analysis revealed distinct physiologic and anesthetic profiles associated with FP tcMEP alerts compared to TP and TN. FP alerts showed significantly lower BIS values and more frequent EEG suppression, consistent with deeper anesthesia. MAP was highest during TP, lowest during TN, and intermediate during FP events, suggesting perfusion alone did not explain signal loss. The elevated MAPs observed during positive alerts were likely a response by the surgical team aimed at increasing spinal cord perfusion pressure, and therefore represent a consequence rather than a confounder of alert status. Propofol and opioid infusion rates did not differ significantly across alert types, but variable sensitivity to anesthetic agents may lead some patients to excessive anesthetic depth. Even under a uniform TIVA protocol, some patients may experience disproportionately deep anesthesia at standard infusion rates, potentially predisposing them to cortical suppression and FP alerts. This suggests that anesthetic depth and cortical suppression, rather than dosage, were the primary factors distinguishing FP events. Temperature was also significantly lower during FP alerts, potentially contributing to altered neuromonitoring responsiveness. Hypothermia may potentiate anesthetic effects on cortical excitability and lower the threshold for EEG suppression [15–17].

Previous work has established that tcMEP amplitude is highly sensitive to anesthetic technique and systemic physiology. TIVA with propofol and short-acting opioids is recommended to preserve MEP integrity during spine deformity surgery [18, 19]. While TIVA is necessary for reliable IONM, less attention has been given to how anesthetic depth, beyond agent choice, affects tcMEP reliability. Studies have reported that excessive cortical suppression, reflected by BIS < 40 and prolonged IBI, is associated with reduced tcMEP reliability [20]. These findings are often based on adult populations or case averages, rather than alert-specific analysis. In a recent pediatric study, Dulfer et al. (2024) found that BIS suppression and prolonged interburst intervals correlated with reduced tcMEP amplitudes, linking deeper anesthesia to diminished neurophysiologic responsiveness. Their mixed cohort analyzed averaged amplitude changes rather than alert classifications and lacked outcome correlation [8].

Hudec et al. (2024) found no consistent association between BIS values and tcMEP reliability in a mixed pediatric and young adult cohort [21]. Their retrospective design, variable anesthetic techniques, and lack of synchronized EEG-MEP integration may explain the discrepancy. By focusing on real-time timepoint-level data under standardized TIVA, our study better isolates cortical suppression (rather than propofol dose or agent choice) as a primary contributor to false positive alerts, building on prior reports linking higher propofol concentrations and volatile anesthesia to tcMEP suppression [22, 23].

BIS is an EEG-derived index of anesthetic depth (0 = isoelectric, 100 = awake) influenced by burst suppression and closely correlated with IBI duration, which quantifies suppressed EEG activity between bursts [10]. BIS values below 40 typically represent deep anesthesia, and longer IBI durations reflect greater cortical inactivation [24]. While BIS does not directly measure spinal cord function, it serves as a real-time marker of anesthetic burden on the central nervous system, and its role in guiding intraoperative neuromonitoring interpretation is increasingly recognized [25–27]. In our study, although average propofol infusion rate did not differ between groups, BIS values in the FP group were significantly lower than in TP and TN events, and EEG suppression (IBI > 0) was almost exclusively observed during FP alerts. In our experience, burst suppression during an alert has proven highly specific for a false positive to the extent that the surgical team has begun to discount these alerts and proceed without intervention when burst suppression is clearly present. These findings suggest that real-time cortical monitoring may add discriminatory power (high specificity) when assessing tcMEP alerts intraoperatively. However, the absence of burst suppression does not necessarily confirm that an alert is genuine. Patients may still experience FP tcMEP changes under deep anesthesia with low BIS values that do not reach the severity required to produce burst suppression. Conversely, the absence of EEG suppression and maintenance of BIS > 50 during TP events may help support the validity of tcMEP signal changes when a surgical insult is suspected.

ROC analysis demonstrated BIS discrimination between FP and TP alerts (AUC 0.924), with a threshold of ≤ 44 yielding 87.5% sensitivity and 88.1% specificity. However, this threshold would still miss 1 in 8 true positives, emphasizing that BIS must be interpreted within surgical context. During routine or low-risk phases, anesthetic optimization may be appropriate, whereas during high-risk corrective maneuvers, tcMEP changes should prompt immediate reassessment regardless of BIS suppression. Notably, no true-positive alerts occurred with BIS values < 34, raising the possibility that such deep suppression may serve as a practical intraoperative rule-out threshold, though this warrants further validation. IBI alone demonstrated limited discriminatory value, with AUC near 0.55, likely due to physiologic collinearity with BIS suppression. BIS alone may serve as a practical, real-time predictor of FP alerts under standardized TIVA conditions.

Anesthetic-related FP alerts caused measurable operative disruption. Six cases were delayed by an average of 14.5 min due to BIS/EEG suppression misinterpreted as surgical injury. One case was aborted entirely, and another was nearly canceled. As the study progressed, team recognition of this anesthetic signature reduced unnecessary responses, underscoring the value of proactive communication between surgical, anesthesia, and neuromonitoring teams.

We recommend establishing shared anesthetic depth targets prior to incision: BIS 50–60 during exposure and closure, and 40–50 during instrumentation and correction. When BIS < 40 with concurrent burst suppression is present at the time of a tcMEP alert, an anesthetic etiology should be strongly considered and anesthetic lightening prioritized, while recognizing that true neurologic injury remains possible and that the surgical cause should not be dismissed without careful clinical assessment. Prospective evaluation of this framework is a priority for future study.

This study has several limitations. The sample size was small, limiting generalizability. Alert classification relied on real-time intraoperative judgment, which introduces inherent subjectivity; however, formal blinding was neither feasible nor ethical in this setting, and no superior classification method exists for alerts occurring during active surgery. Because determinations were made prospectively based on surgical context alone, prior to any rescue intervention, rescue maneuvers are unlikely to have influenced alert classification. Separately, timepoint-level analyses treat repeated measures as independent observations, potentially inflating statistical significance due to within-patient clustering. Given the small cohort and imbalanced group sizes, mixed-effects modeling was not appropriate; the large effect sizes and near-complete EEG suppression separation suggest findings are robust, though multicenter validation is necessary*.* Although all cases followed a standardized anesthetic protocol, variability in pharmacodynamics may have influenced results. Propofol infusion rates were recorded separately from neuromonitoring data, so rapid changes may not have been captured. Serum anesthetic levels were not obtained; future studies incorporating pharmacokinetic data would better characterize the relationship between anesthetic exposure, cortical suppression, and tcMEP reliability.

Despite these limitations, the study has important clinical implications. By identifying EEG suppression and low BIS as common features of FP tcMEP alerts, these data highlight the potential for enhanced intraoperative decision-making using multimodal monitoring. Real-time assessment of anesthetic depth may help reduce unnecessary interruptions, minimize operative delays, and improve confidence when assessing the clinical significance of tcMEP changes, especially in the absence of obvious surgical cause.

In summary, our findings suggest that FP tcMEP alerts in pediatric spine surgery are associated with deeper anesthesia and cortical suppression, despite stable anesthetic dosing. Integrating EEG and BIS monitoring into neuromonitoring response workflows may improve the accuracy of tcMEP interpretation, reduce unnecessary surgical responses to FP alerts, and ultimately enhance the safety and efficiency of pediatric spinal deformity surgery. This study represents an initial step toward a validated framework for interpreting tcMEP alerts in pediatric deformity surgery; next steps include external multicenter validation with streamlined prospective data collection and evaluation of workflow enhancements integrating processed EEG/BIS into alert management, while mechanistic work in controlled animal models may further clarify anesthetic-driven tcMEP suppression.

## Data Availability

The datasets generated and analyzed during the current study are not publicly available due to institutional data privacy restrictions and the inclusion of protected health information from pediatric patients but may be made available from the corresponding author on reasonable request and with appropriate institutional approval.
